# Mode of antiviral action of silver nanoparticles against HIV-1

**DOI:** 10.1186/1477-3155-8-1

**Published:** 2010-01-20

**Authors:** Humberto H Lara, Nilda V Ayala-Nuñez, Liliana Ixtepan-Turrent, Cristina Rodriguez-Padilla

**Affiliations:** 1 Laboratorio de Inmunología y Virología, Departamento de Microbiología e Inmunología, Facultad de Ciencias Biologicas, Universidad Autonoma de Nuevo Leon, San Nicolas de los Garza, Mexico

## Abstract

**Background:**

Silver nanoparticles have proven to exert antiviral activity against HIV-1 at non-cytotoxic concentrations, but the mechanism underlying their HIV-inhibitory activity has not been not fully elucidated. In this study, silver nanoparticles are evaluated to elucidate their mode of antiviral action against HIV-1 using a panel of different *in vitro *assays.

**Results:**

Our data suggest that silver nanoparticles exert anti-HIV activity at an early stage of viral replication, most likely as a virucidal agent or as an inhibitor of viral entry. Silver nanoparticles bind to gp120 in a manner that prevents CD4-dependent virion binding, fusion, and infectivity, acting as an effective virucidal agent against cell-free virus (laboratory strains, clinical isolates, T and M tropic strains, and resistant strains) and cell-associated virus. Besides, silver nanoparticles inhibit post-entry stages of the HIV-1 life cycle.

**Conclusions:**

These properties make them a broad-spectrum agent not prone to inducing resistance that could be used preventively against a wide variety of circulating HIV-1 strains.

## Background

According to the Joint United Nations Programme on HIV/AIDS, an estimated 33 million people were living with HIV in 2007, 2.7 million fewer than in 2001 [1]. Although the rate of new HIV infections has fallen in several countries, the HIV/AIDS pandemic still stands as a serious public health problem worldwide. The emergence of resistant strains is one of the principal challenges to containing the spread of the virus and its impact on human health. In different countries, studies have shown that 5%-78% of treated patients receiving antiretroviral therapy are infected with HIV-1 viruses that are resistant to at least one of the available drugs [2]. For these reasons, there is a need for new anti-HIV agents that function over viral stages other than retrotranscription or protease activity and that can be used for treatment and prevention of HIV/AIDS dissemination [3].

Fusion or entry inhibitors are considered an attractive option, since blocking HIV entry into its target cell leads to suppression of viral infectivity, replication, and the cytotoxicity induced by the virus-cell interaction [4]. Since 2005, only two fusion inhibitors have been approved by the FDA (Enfurtivide and Maravirovic).

In addition to fusion inhibitors, virucidal agents are urgently needed for HIV/AIDS prevention because they directly inactivate the viral particle (virion), which prevents the completion of the viral replication cycle. Virucidal agents differ from virustatic drugs in that they act directly and rapidly by lysing viral membranes on contact or by binding to virus coat proteins [5]. These compounds would directly interact with HIV-1 virions to inactivate infectivity or prevent infection and could be used as an approach to provide a defense against sexual transmission of the virus [6].

Previously, we explored the antiviral properties of silver nanoparticles against HIV-1 and found by *in vitro *assays that they are active against a laboratory-adapted HIV-1 strain at non-cytotoxic concentrations. Images obtained by high angle annular dark field (HAADF) scanning transmission electron microscopy (STEM) show gp120 as its possible molecular target. Using this technique, a regular spatial arrangement of the silver nanoparticles attached to HIV-1 virions was observed. The center-to-center distance between the silver nanoparticles (~28 nm) was similar to the spacing of gp120 spikes over the viral membrane (~22 nm). It was hypothesized that the exposed sulfur-bearing residues of the glycoprotein knobs would be attractive sites for nanoparticle interaction [7]. However, the mechanism underlying the HIV-inhibitory activity of silver nanoparticles was not fully elucidated.

Nanotechnology offers opportunities to re-explore biological properties of known antimicrobial compounds by manipulation of their sizes. Silver has long been known for its antimicrobial properties, but its medical applications declined with the development of antibiotics. Nonetheless, Credés prophylaxis of gonococcal *ophthalmia neonatorum *remained the standard of care in many countries until the end of the 20^th ^century [8]. Currently, silver sulfadiazine is listed by the World Health Organization as an essential anti-infective topical medicine [9]. Silver's mode of action is presumed to be dependent on Ag^+ ^ions, which strongly inhibit bacterial growth through suppression of respiratory enzymes and electron transport components and through interference with DNA functions [10]. If silver as a bulk material works, would nano-size silver be appealing? In medicine, the potential of metal nanoparticles has been explored for early detection, diagnosis, and treatment of diseases, but their biological properties have largely remained unexplored [11].

Silver nanoparticles have been studied for their antimicrobial potential and have proven to be antibacterial agents against both Gram-negative and Gram-positive bacteria [12-16], and antiviral agents against the HIV-1 [17] hepatitis B virus [18] respiratory syncytial virus [19] herpes simplex virus type 1 [20] and monkeypox virus [21]. The development of silver nanoparticle products is expanding. They are now used as part of clothing, food containers, wound dressings, ointments, implant coatings, and other items [22,23]; some silver nanoparticle applications have received approval from the US Food and Drug Administration [24].

To better understand the mode of action by which silver nanoparticles inactivate HIV-1 and their potential as a virucidal agent, we used a panel of assays that included: (*i*) a challenge against a panel of various HIV-1 strains, (*ii*) virus adsorption assays, (*iii*) cell-based fusion assays, (*iv*) a gp120/CD4 capture ELISA, (*v*) time-of-addition experiments, (*vi*) virucidal activity assays with cell-free virus, and (*vii*) a challenge against cell-associated virus. The data from these experiments suggest that silver nanoparticles exerted anti-HIV activity at an early stage of viral replication, most likely as a virucidal agent or viral entry inhibitor.

## Results

### Cytotoxic effect

HeLa-CD4-LTR-β-gal cells (which express both CXCR4 and CCR5), MT-2 cells (lymphoid human cell line expressing CXCR4), and human PBMC, were used as models to assess silver nanoparticles' cytotoxicity. By means of a luciferase-based assay, the 50% cytotoxic concentration (CC_50_) of silver nanoparticles was defined as 3.9 ± 1.6 mg/mL against HeLa-CD4-LTR-β-gal cells, as 1.11 ± 0.32 mg/mL against human PBMC, and 1.3 ± 0.58 mg/mL against MT-2 cells.

### Range of antiviral activity

Silver nanoparticles of 30-50 nm were tested against a panel of HIV-1 isolates using indicator cells in which infection was quantified by a luciferase-based assay. Silver nanoparticles inhibited all strains, showing comparable antiviral potency against T-tropic, M-tropic, dual-tropic, and resistant isolates (Table 1). The concentration of silver nanoparticles at which infectivity was inhibited by 50% (IC_50_) ranged from 0.44 to 0.91 mg/mL. The therapeutic index reflects a compound's overall activity by relating cytotoxicity (CC_50_) and effectiveness, measured as the ability to inhibit infection (IC_50_), under the same assay conditions. For these strains of HIV-1, no significant reduction of the therapeutic index was observed in strains that were resistant toward NNRTI, NRTI, PI, and PII compared with laboratory strains catalogued as wild type virus (Table 1).

**Table 1:** Antiviral effect of silver nanoparticles against HIV-1 strains

HIV-1 strain	Tropism (co-receptor)	IC_50 _(mg/mL)*	HeLa cells CC_50 _(mg/mL)*	TI
IIIB	T (X4)	0.44 (± 0.3)	3.9 (± 1.6)	8.9
Eli	T (X4)	0.42 (± 0.2)		9.3
Beni	T (X4)	0.19 (± 0.1)		20.5
96USSN20	T (X4)/M (R5)	0.36 (± 0.2)		12.5
Bal	M (R5)	0.27 (± 0.2)		14.4
BCF01	M (R5)	0.37 (± 0.3)		10.5
AZT_RV_	T (X4)	0.19 (± 0.01)		20.5
NNRTI_RV_	T (X4)	0.61 (± 0.24)		6.4
PI_RV_	T (X4)	0.91 (± 0.09)		4.3
3TC_RV_	T (X4)	0.73 (± 0.12)		5.3
Saquinavir_RV_	T (X4)	0.81 (± 0.11)		4.8

*Values represent the mean of the triplicate ± standard error of the mean.

NNRTI: non-nucleoside retrotranscriptase inhibitor, PI: protease inhibitor, RV: resistant virus

### Antiviral activity of silver nanoparticles and ions

To define that the observed antiviral effect of silver nanoparticles is due to nanoparticles, rather than just silver ions present in the solution, we also assessed the antiviral activity of silver sulfadiazine (AgSD) and silver nitrate (AgNO_3_), known antimicrobial silver salts that exert their antimicrobial effect through silver ions [25]. Both salts inhibited HIV-1 infection *in vitro *(Table 2), however, their therapeutic index is 12 times lower than the one of silver nanoparticles, which indicates that silver ions by itself have a lower efficiency than silver nanoparticles.

**Table 2:** Antiviral effect of silver salts and nanoparticles against HIV-1

Silver compound	IC_50_*	HeLa cells CC_50_*	TI
Silver nanoparticles	0.44 mg/mL (± 0.3)	3.9 mg/mL (± 1.6)	8.9
Silver sulfadiazine	39.33 μg/mL (± 14.60)	28.25 μg/mL (± 7.28)	0.7
Silver nitrate	0.00059% (± 0.00022%)	0.00044% (± 0.00002%)	0.7

*Values represent the mean of the triplicate ± standard error of the mean.

### Inhibition of viral adsorption

To confirm that the anti-HIV activity of silver nanoparticles can be attributed to the inhibition of virus binding or fusion to the cells, a virus adsorption assay was performed [26]. One fusion inhibitor (Enfuvirtide) was included as control specimen. Silver nanoparticles inhibited the binding of IIIB virus to cells with an IC_50 _of 0.44 mg/mL. As expected, the fusion inhibitor inhibited virus adsorption. These results indicate that silver nanoparticles inhibit the initial stages of the HIV-1 infection cycle.

### Inhibition of Env/CD4-mediated membrane fusion

**Figure 1:**
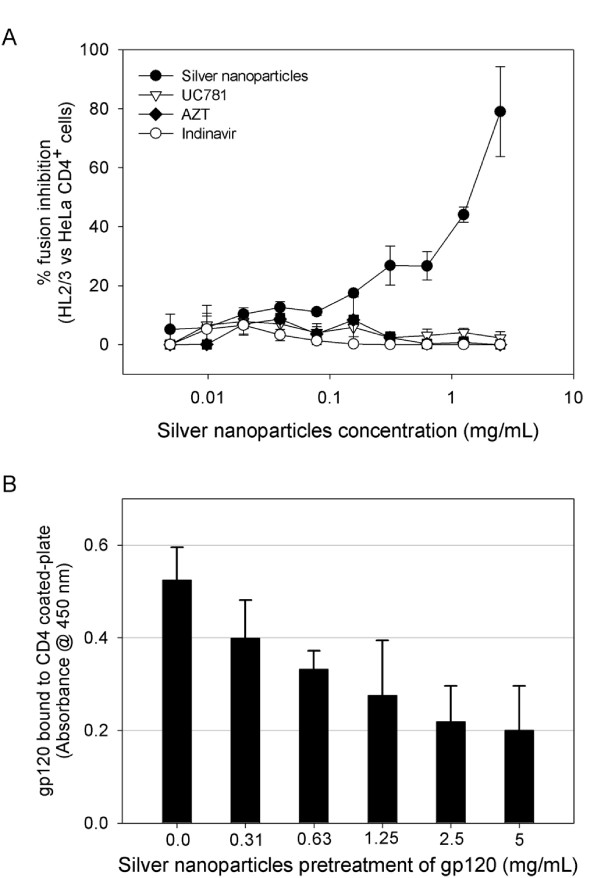
**Inhibition of the gp120-CD4 interaction**. (A) A cell-based fusion assay was used to mimic the gp120-CD4 mediated fusion of the viral and host cell membranes. HL2/3 and HeLa-CD4-LTR-β-gal cells were incubated with a two-fold serial dilution of silver nanoparticles and known antiretrovirals. The assay was performed in triplicate; the data points represent the mean ± s.e.m. (B) The degree of inhibition of the gp120-CD4 protein binding was assessed with a gp120/CD4 ELISA capture in the presence or absence of silver nanoparticles. Gp120 protein was pretreated for 10 min with a two-fold serial dilution of silver nanoparticles, then added to a CD4-coated plate. The assay was done twice; the error bars indicate the s.e.m.

A cell-based fusion assay was used to mimic the gp120-CD4-mediated fusion process of HIV-1 to the host cell. HL2/3 cells, which express HIV-1 Env on their surfaces and Tat protein in their cytoplasms (effector cells) [27] and HeLa-CD4-LTR-β-gal (indicator cells) can fuse as the result of the gp120-CD4 interaction, and the amount of fused cells can be measured with the β-gal reporter gene. In the presence of a HL2/3-HeLa CD4 mixture, silver nanoparticles efficiently blocked fusion between both cells (Figure 1A) in a dose-dependent manner (1.0-2.5 mg/mL range). This concentration range is close to what we previously reported for silver nanoparticles IC_50_. Known antiretroviral drugs used as controls, such as UC781 (NNRTI), AZT (NRTI), and Indinavir (PI), did not inhibit cell fusion in this cell-based fusion assay.

### Silver nanoparticles interfere with gp120-CD4 interaction

The inhibitory activity of silver nanoparticles against the gp120-CD4 interaction was also investigated in a competitive gp120-capture ELISA. A constant amount of gp120 was incubated for 10 min with increasing amounts of silver nanoparticles, the mixture was then added to a CD4-coated plate, and the amount of gp120 bound to the plate was quantified. Compared with the control (0.0 mg/mL), there was a decrease of over 60% of gp120 bound to CD4 coated-plates at the highest dose of silver nanoparticles. As shown in Figure 1B, significant decreases in absorbance values were observed in the presence of silver nanoparticles (0.3-5.0 mg/mL). The gp120-capture ELISA data, combined with the results of the cell-based fusion assay, support the hypothesis that silver nanoparticles inhibit HIV-1 infection by blocking the viral entry, particularly the gp120-CD4 interaction.

Although silver nanoparticles feature characteristic absorption at 400-500 nm [28] no interference to the absorption signals of the ELISA assay was observed. This can be assumed since the wells with the highest concentration of silver nanoparticles did display higher absorption levels (see Figure 1B) than the controls (0.0 mg/mL). Besides, the absorption levels obtained in the presence of silver nanoparticles were lower than the ones of the calibration curve (as defined by the manufacturer).

### Time (Site) of Intervention

**Figure 2:**
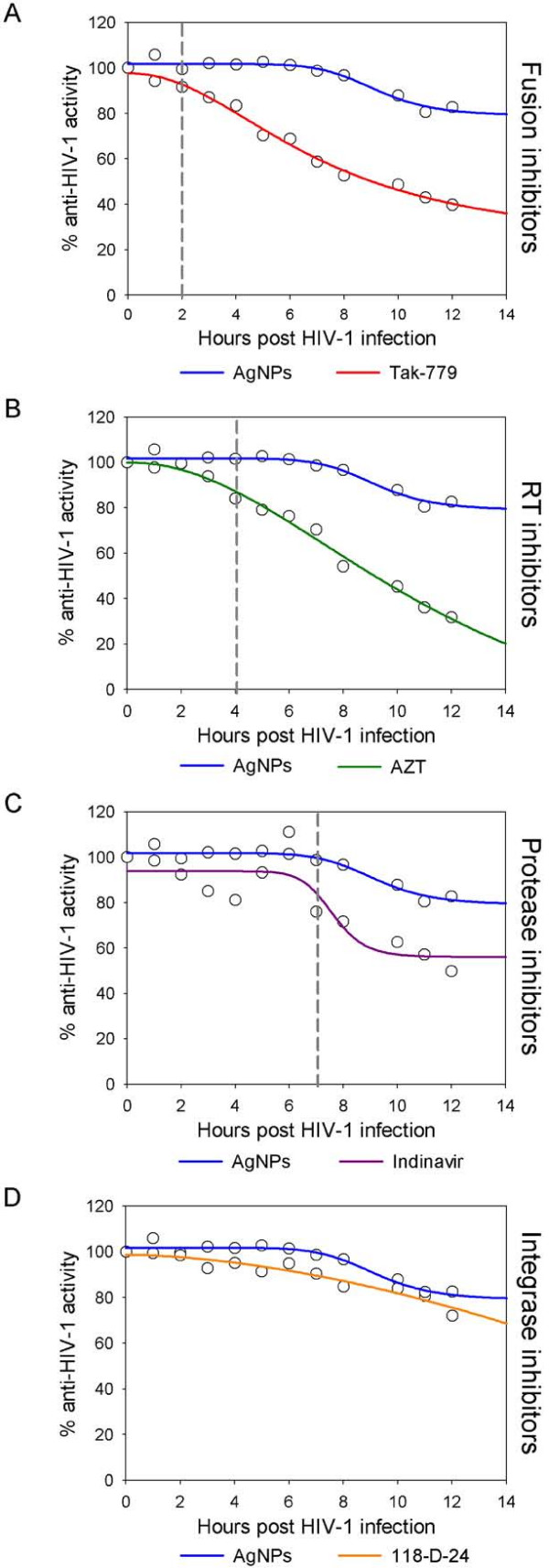
**Time-of-addition experiment**. HeLa-CD4-LTR-β-gal cells were infected with HIV- 1_IIIB_, and silver nanoparticles (1 mg/mL) and different antiretrovirals were added at different times post infection. Activity of silver nanoparticles was compared with (A) Fusion inhibitors (Tak-779, 2 μM), (B) RT inhibitors (AZT, 20 μM), (C) Protease inhibitors (Indinavir, 0.25 μM), and (D) Integrase inhibitors (118-D-24, 100 μM). Dashed lines indicate the moment when the activity of the silver nanoparticles and the antiretroviral differ. The assay was performed in triplicate; the data points represent the mean and the colored lines are nonlinear regression curves done with SigmaPlot 10.0 software.

To further determine the antiviral target of silver nanoparticles, a time-of-addition experiment was performed using a single cycle infection assay. The time-of-addition experiment was used to delimit the stage(s) of the viral life cycle that is blocked by silver nanoparticles. HeLa cells (expressing CD4, CXCR4 and CCR5) were infected with HIV-1_IIIB _cell-free virus and either silver nanoparticles (1.0 mg/mL), Tak-779 (2.0 μM), AZT (20.0 μM), Indinavir (0.25 μM), or 118-D-24 (100.0 μM) was added upon HIV-1 inoculation (time zero) or at various time points post-inoculation. These antiretroviral drugs were chosen as controls as they point out different stages of the viral cycle (fusion or entry, retrotranscription, protease activity, and integration to the genome). As seen in Figure 2(A-D), the antiviral activity of Tak-779, AZT, Indinavir, and 118-D-24 started to decline after the cycle stage that they target has passed. The fusion inhibitor's activity declined after 2 h (Figure 2A), RT inhibitors after 4 h (Figure 2B), protease inhibitors after 7 h (Figure 2C), and integrase inhibitors after 12 h (Figure 2D). In contrast, silver nanoparticles retained their antiviral activity even when added 12 h after the HIV inoculation. These results show that silver nanoparticles intervene with the viral life cycle at stages besides fusion or entry. These post-entry stages cover a time period between and including viral entry and the integration into the host genome.

### Virucidal activity of silver nanoparticles: inactivation of cell-free and cell-associated virus

**Figure 3:**
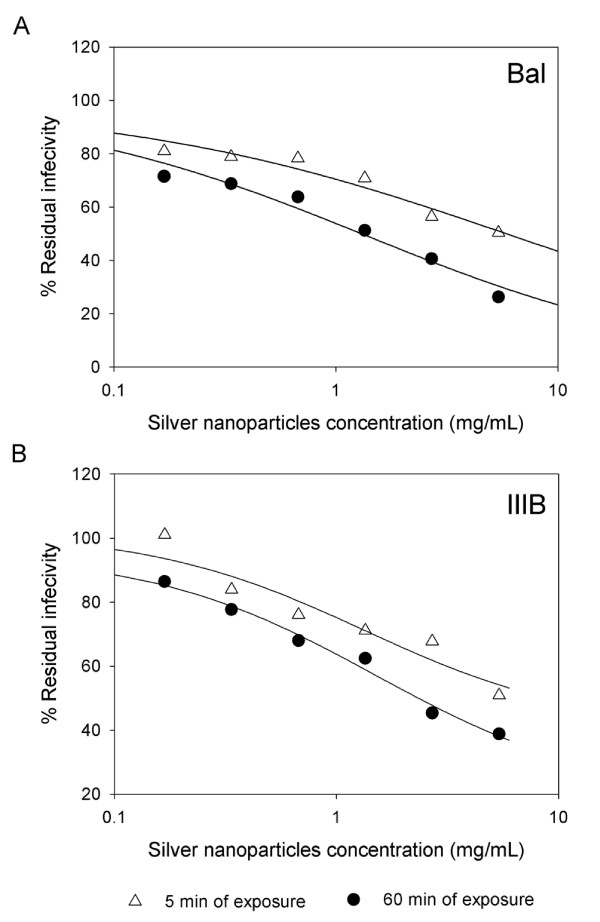
**Virucidal activity of silver nanoparticles against M and T tropic HIV-1**. Serial two-fold dilutions of silver nanoparticles were added to 10^5 ^TCID_50 _of HIV-1_Bal _(A) and HIV-1_IIIB _(B) cell-free virus with a 0.2-0.5 m.o.i. After incubation for 5 min and 60 min, the mixtures were centrifuged three times at 10,000 rpm, the supernatant fluids removed, and the pellets washed three times. The final pellets were placed into 96-well plates with HeLa-CD4-LTR-β-gal cells. Assessment of HIV-1 infection was made with a luciferase-based assay. The percentage of residual infectivity after silver nanoparticle treatment was calculated with respect to the positive control of untreated virus. The assay was performed in triplicate; the data points represent the mean, and the solid lines are nonlinear regression curves done with SigmaPlot 10.0 software.

To study the effect that silver nanoparticles have over the virus itself, cell-free and cell-associated HIV-1 were treated with different concentrations of nanoparticles. Cell-free and cell-associated virus are the infectious HIV-1 forms present in semen and cervicovaginal secretions and can be transmitted across the mucosal barrier [29] Cell-associated virus includes infected cells that transmit the infection by fusing with non-infected receptor cells. By means of a luciferase-based assay, the residual infectivity of cell-free viruses (one T-tropic and one M-tropic) was quantified after silver nanoparticle treatment. As shown in Figure 3(A-B), silver nanoparticle pretreatment of HIV-1_IIIB _and HIV-1_Bal _decreased the infectivity of the viral particles after just 5 min of exposure. The effect increased after 60 min of exposure (particularly in Bal), indicating that silver nanoparticles act directly on the virion, inactivating it.

**Figure 4:**
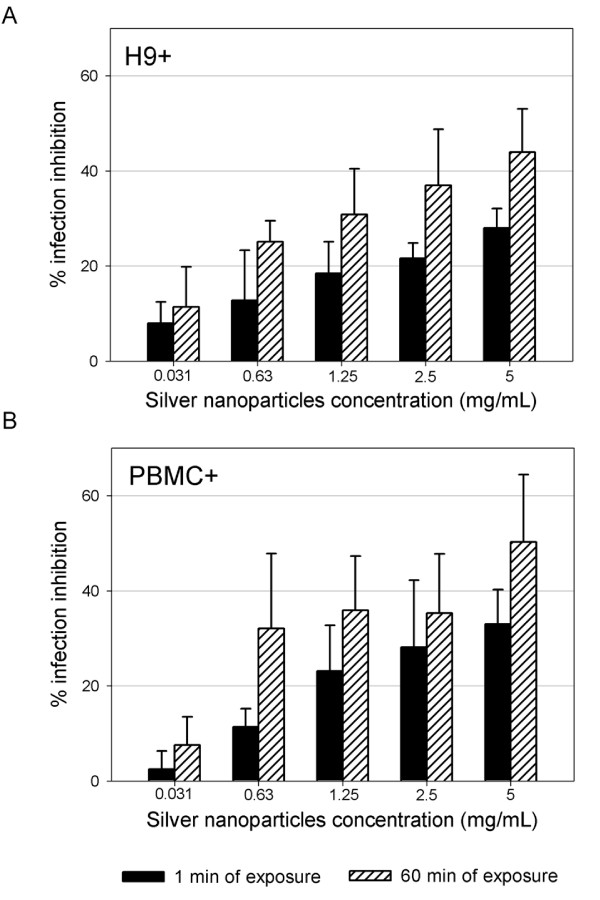
**Treatment of HIV-1 cell-associated virus**. Chronically HIV-1-infected H9 (A) and PBMC (B) cells were incubated with serial two-fold dilutions of silver nanoparticles for 1 min and 60 min. Treated cells were centrifuged, washed three times with cell culture media, and then added to TZM-bl cells. Assessment of HIV-1 infection was made with a luciferase-based assay after 48 h. The assay was performed in triplicate; the error bars indicate the s.e.m.

Silver nanoparticles were also effective against the transmission of HIV-1 infection mediated by chronically infected PBMC and H9 (human lymphoid cell line). Transmission was 50% reduced, even when both cell types were treated with the nanoparticles for 1 min (Figure 4A-B).

## Discussion

Silver nanoparticles proved to be an antiviral agent against HIV-1, but its mode of action was not fully elucidated. Is gp120 its principal target? Do silver nanoparticles act as entry inhibitors? In this study, we investigated the mode of antiviral action of silver nanoparticles against HIV-1. Our results reveal, for the first time, that silver nanoparticles exert anti-HIV activity at an early stage of viral replication, most likely as a virucidal agent or viral entry inhibitor.

No significant difference was found in the antiviral activities of silver nanoparticles against the different drug-resistant strains (Table 1), so the mutations in antiretroviral HIV strains that confer resistance do not affect the efficacy of silver nanoparticles. These results further agree with previous findings, where it was proven that silver nanoparticles are broad-spectrum biocides [30,31] HIV-1 strains found in the human population can differ widely in their pathogenicity, virulence, and sensitivity to particular antiretroviral drugs [32] The fact that silver nanoparticles inhibit such a varied panel of strains makes them an effective broad-spectrum agent against HIV-1. This particular property can reduce the likelihood of the emergence of resistance and the subsequent spread of infection.

Silver nanoparticles inhibited a variety of HIV-1 strains regardless of their tropism (Table 1). Variation in gp120 among HIV strains is the major determinant of differing tropism among strains, with the V3 loop of gp120 recognizing the chemokine receptors CXCR4 (T-tropic virus), CCR5 (M-tropic virus), or both (dual-tropic virus) [33] The fact that silver nanoparticles inhibited all tested strains indicates that their mode of action does not depend on this determinant of cell tropism. Elechiguerra *et al*. postulated that silver nanoparticles undergo specific interaction with HIV-1 via preferential binding with gp120 [7] If so, then our findings show that inhibition by silver nanoparticles is not dependent on the V3 loop, which has a net positive charge that contributes to its role in determining viral co-receptor tropism [34] Since silver particles have a positive surface charge, the V3 loop would not be their preferred site of interaction. Hence, the nanoparticles may possibly act as attachment inhibitors by impeding the gp120-CD4 interaction, rather than as co-receptor antagonists that interfere with the gp120-CXCR4/CCR5 contact [4]

By means of a viral adsorption assay, it was shown that silver nanoparticles' mechanism of anti-HIV action is based on the inhibition of the initial stages of the HIV-1 cycle. In addition, the gp120-capture ELISA data (Figure 1B), combined with the results of the cell-based fusion assay (Figure 1A), supported the hypothesis that silver nanoparticles inhibit HIV-1 infection by blocking viral entry, particularly the gp120-CD4 interaction. The observations previously made by STEM analysis support this idea, since silver nanoparticles were seen to bind protein structures distributed over the viral membrane [7] If silver nanoparticles do not bind to the V3 loop, then they might preferentially interact with the negative cavity of gp120 that binds to CD4 [35] The attraction between CD4 and gp120 is mostly electrostatic, with the primary end of CD4 binding in a recessed pocket on gp120, making extensive contacts over ~800 Å^2 ^of the gp120 surface [36]

In addition, silver nanoparticles might interact with the two disulfide bonds located in the carboxyl half of the HIV-1 gp120 glycoprotein, an area that has been implicated in binding to the CD4 receptor [37] Silver ions bind to sulfhydryl groups, which lead to protein denaturation by the reduction of disulfide bonds [38] Therefore, we hypothesize that silver nanoparticles not only bind to gp120 but also modify this viral protein by denaturing its disulfide-bonded domain located in the CD4 binding region. This can be seen in our results of silver nanoparticles' capacity to more strongly diminish residual infectivity of viral particles after 60 minutes of incubation than after 5 minutes of incubation (Figure 3). Since the antiviral effect of silver nanoparticles increases with the incubation time, we can hypothesize that silver nanoparticles initially bind to gp120 knobs and then inhibit infection by irreversibly modifying these viral structures. However, further research is needed to define if silver nanoparticles interact with the negatively charged cavity and the two disulfide bonds located in gp120's CD4 binding region.

Resistance development may be an issue for compounds that target the envelope because of the high rate of substitutions in the variable regions of the Env protein. However, since the positions of the cysteine residues, the disulfide bonding pattern in gp120, and the ability of gpl20 to bind to the viral receptor CD4 are highly conserved between isolates [39] the development of resistance to silver nanoparticles would be complicated.

By comparing the antiviral effect (measured by the therapeutic index) of silver nanoparticles with two commonly used silver salts (AgSD and AgNO_3_), it was observed that silver ions by themselves are less efficient than silver nanoparticles. Hence, if the observed anti-HIV-1 activity of silver nanoparticles would just have been due to silver ions present in the nanoparticles' solution, the therapeutic index would have been lower. High activity of silver nanoparticles is suggested to be due to species difference as they dissolve to release Ag^0 ^(atomic) and Ag^+ ^(ionic) clusters, whereas silver salts release Ag^+ ^only [40]

The time-of-addition experiments further confirmed silver nanoparticles as entry inhibitors (Figure 2). In addition, it was revealed that silver nanoparticles have other sites of intervention on the viral life cycle, besides fusion or entry. Since silver ions can complex with electron donor groups containing sulfur, oxygen, or nitrogen that are normally present as thiols or phosphates on amino acids and nucleic acids [41] they might inhibit post-entry stages of infection by blocking HIV-1 proteins other than gp120, or reducing reverse transcription or proviral transcription rates by directly binding to the RNA or DNA molecules. Besides, earlier studies have shown that silver nanoparticles suppress the expression of TNF-α [42] which is a cytokine that plays a pivotal role in HIV-1 pathogenesis by incrementing HIV-1 transcription [43] The inhibition of the TNF-α activated transcription might also be a target for the anti-HIV activity of silver nanoparticles. Having such a varied panel of targets in the HIV-1 replication cycle makes silver nanoparticles an agent that is not prone to contribute to the appearance of resistant strains.

Silver nanoparticles proved to be virucidal to cell-free and cell-associated HIV-1 as judged by viral infectivity assays (Figures 3 and 4). HIV infectivity is effectively eliminated following short exposure of isolated virus to silver nanoparticles. Silver nanoparticle treatment of chronically infected H9^+ ^cells as well as human PBMC^+ ^resulted in decreased infectivity.

A virucide must operate quickly and effectively in preventing infection of vulnerable target cells. According to Borkow *et al*. (1997), an ideal retrovirucidal agent should act directly on the virus, act at replication steps prior to integration of proviral DNA into the infected host cell genome, be absorbable by uninfected cells in order to provide a barrier to infection by residual active virus, and be effective at non-cytotoxic concentrations readily attainable *in vivo *[44] Silver nanoparticles act directly on the virus at steps that prevent integration inside the host cell, but further pharmacokinetic, pharmacodynamic, and toxicological studies in animal models are needed to define safety parameters for the use of silver nanoparticles as preventive tools for HIV-1 transmission.

## Conclusions

Finally, we propose that the antiviral activity of silver nanoparticles results from their inhibition of the interaction between gp120 and the target cell membrane receptors. According to our results, this mode of antiviral action allows silver nanoparticles to inhibit HIV-1 infection regardless of viral tropism or resistance profile, to bind to gp120 in a manner that prevents CD4-dependent virion binding, fusion, and infectivity, and to block HIV-1 cell-free and cell-associated infection, acting as a virucidal agent. In conclusion, silver nanoparticles are effective virucides as they inactivate HIV particles in a short period of time, exerting their activity at an early stage of viral replication (entry or fusion) and at post-entry stages. The data presented here contribute to a new and still largely unexplored area; the use of nanomaterials against specific targets of viral particles.

## Methods

### Silver compounds

Commercially manufactured 30-50 nm silver nanoparticles, surface coated with 0.2 wt% PVP, were used (Nanoamor, Houston, TX). Stock solutions of silver nanoparticles, silver sulfadiazine (Sigma-Aldrich) and silver nitrate (Sigma-Aldrich) were prepared in RPMI 1640 cell culture media. Following serial dilutions of the stock were made in culture media.

### Cells, HIV-1 isolates, and antiretrovirals

HeLa-CD4-LTR-β-gal cells, MT-2 cells, HL2/3 cells, H9 cells, TZM-bl cells, HIV-1_IIIB_, HIV-1_Bal_, HIV-1_BCF01_, HIV-1_96USSN20_, AZT, Indinavir, 118-D-24, Tak-779, and Enfuvirtide were obtained through the AIDS Research and Reference Reagent Program, NIH. HIV-1_Eli _and HIV-1_Beni _are clinical isolates from patients from the Ruth Ben-Ari Institute of Clinical Immunology and AIDS Center, Israel. They were kindly donated by Gadi Borkow. Aliquots of cell-free culture viral supernatants were used as viral inocula. Peripheral blood mononuclear cells (PBMC) were isolated from healthy donors using Histopaque-1077 (Sigma-Aldrich) according to the manufacturer's instructions. UC781 was kindly donated by Dr. Gadi Borkow.

### Cytotoxicity assays

A stock solution of silver nanoparticles was two-fold diluted to desired concentrations in growth medium and subsequently added into 96-wells plates containing HeLa-CD4-LTR-β-gal cells, PBMC and MT-2 cells (5 × 10^4 ^cells/well). Microtiter plates were incubated at 37°C in a 5% CO_2 _air humidified atmosphere for a further 2 days. Assessments of cell viability were carried out using a CellTiter-Glo^® ^Luminescent Cell Viability Assay (Promega). The 50% cytotoxic concentration (CC_50_) was defined based on the percentage cell survival relative to the positive control.

### HIV-1 infectivity inhibition assays

Serial two-fold dilutions of silver nanoparticles were mixed with 10^5 ^TCID_50 _of HIV-1 cell-free virus and added to HeLa-CD4-LTR-β-gal cells with a 0.2-0.5 multiplicity of infection [7] HIV-1 infection was assessed after two days of incubation by quantifying the activity of the β-galactosidase produced after infection with the Beta-Glo Assay System (Promega). The 50% inhibitory concentration (IC_50_) was defined according to the percentage of infectivity inhibition relative to the positive control.

### Virus adsorption assays

In this assay the inhibitory effects of silver nanoparticles on virus adsorption to HeLa-CD4-LTR-β-gal cells were measured as previously described [26] HeLa-CD4-LTR-β-gal cells (5 × 10^4 ^cells/well) were incubated with HIV_IIIB _in the absence or presence of serial dilutions of silver nanoparticles and Enfuvirtide. After 2 h of incubation at 37°C, the cells were extensively washed with 1× PBS to remove the unadsorbed virus particles. Then the cells were incubated for 48 h, and the amount of viral infection was quantified with the Beta-Glo Assay System (Promega).

### Cell-based fusion assay

HeLa-derived HL2/3 cells, which express the HIV-1_HXB2 _Env, Tat, Gag, Rev, and Nef proteins, were co-cultured with HeLa-CD4-LTR-β-gal cells at a 1:1 cell density ratio (2.5 × 10^4 ^cells/well each) for 48 h in the absence or presence of two-fold dilutions of silver nanoparticles, UC781, AZT, and Indinavir in order to examine whether the compounds interfered with the binding process of HIV-1 Env and the CD4 receptor. Upon fusion of both cell lines, the Tat protein from HL2/3 cells activates β-galactosidase indicator gene expression in HeLa-CD4-LTR-β-gal cells [45,27] β-gal activity was quantified with the Beta-Glo Assay System (Promega). The percentage of inhibition of HL2/3-HeLa CD4 cell fusion was calculated with respect to the positive control of untreated cells.

### HIV-1 gp120/CD4 ELISA

A gp120 capture ELISA (ImmunoDiagnostics, Inc., Woburn, MA) was used to test the inhibitory activity of silver nanoparticles against gp120-CD4 binding. Briefly, recombinant HIV-1_IIIB _gp120 protein (100 ng/mL) was pre-incubated for 10 min in the absence or presence of serial two-fold dilutions of silver nanoparticles, and then added to a CD4-coated plate. The amount of captured gp120 was detected by peroxidase-conjugated murine anti-gp120 MAb. In separate experiments, gp120 (100 ng/mL) was added to CD4-coated plates pretreated with silver nanoparticles for a 10 min period. Before the addition of the gp120 protein, plates were washed three times to remove unbound silver nanoparticles [27]

### Time-of-addition experiments

HeLa-CD4-LTR-β-gal cells were infected with 10^5 ^TCID_50 _of HIV-1 cell-free virus with a 0.2-0.5 multiplicity of infection (m.o.i.). Silver nanoparticles (1 mg/mL), Tak-779 (fusion inhibitor, 2 μM), AZT (NRTI, 20 μM), Indinavir (protease inhibitor, 0.25 μM), and 118-D-24 (integrase inhibitor, 100 μM) were then added at different times (0, 1, 2, 3 ... 12 h) after infection [3,31] Infection inhibition was quantified after 48 h by measuring β-gal activity with the Beta-Glo Assay System.

### Virucidal activity assay

Serial two-fold dilutions of silver nanoparticles were added to 10^5 ^TCID_50 _of HIV-1_IIIB _and HIV-1_Bal _cell-free virus with a 0.2-0.5 m.o.i. After incubation for 5 min and 60 min at room temperature, the mixtures were centrifuged three times at 10,000 rpm, the supernatant fluids removed, and the pellets washed three times. The final pellets were resuspended in DMEM and placed into 96-well plates with HeLa-CD4-LTR-β-gal cells. The cells were incubated in a 5% CO_2 _humidified incubator at 37°C for 2 days. Assessment of HIV-1 infection was made with the Beta-Glo Assay System. The percentage of residual infectivity after silver nanoparticle treatment was calculated with respect to the positive control of untreated virus [31]

### Treatment of HIV-1 cell-associated virus

Chronically HIV-1-infected PBMC and H9 cells were incubated with serial two-fold dilutions of silver nanoparticles for 1 min and 60 min. Treated cells were centrifuged, washed three times with cell culture media, and then added to TZM-bl cells. HIV-1 infection triggers, through the Tat protein, β-galactosidase expression in TZM-bl cells. β-gal activity was quantified with the Beta-Glo Assay System.

### Statistical analysis

Graphs show values of the means ±standard deviations from three separate experiments, each of which was carried out in duplicate. Time-of-addition experiment graphs are nonlinear regression curves done with SigmaPlot 10.0 software.

## Competing interests

The authors declare that they have no competing interests.

## Authors' contributions

All authors read and approved the final manuscript. HHL participated in the conception and experimental design of the *in vitro *HIV-1 manipulation and infectivity assays, in analysis and interpretation of the data, and in writing and revision of this report. NVAN. participated in the conception and design of the *in vitro *HIV-1 manipulation and infectivity assays, in analysis and interpretation of the data, and in writing and revision of this report. LIT participated in collection of *in vitro *HIV-1 manipulation and infectivity assays. C.R-P. participated in the experimental design of this research.
